# Rhizobacteria Isolated from Amazonian Soils Reduce the Effects of Water Stress on the Growth of Açaí (*Euterpe oleracea* Mart.) Palm Seedlings

**DOI:** 10.3390/biology13100757

**Published:** 2024-09-24

**Authors:** Suania Maria do Nascimento Sousa, Josinete Torres Garcias, Marceli Ruani De Oliveira Farias, Allana Laís Alves Lima, Rosiane do Socorro dos Reis de Sousa, Hellen Kempfer Philippsen, Lucimar Di Paula dos Santos Madeira, Herve Rogez, Joana Montezano Marques

**Affiliations:** 1 Institute of Biological Sciences, Federal University of Pará, Belém 66075-110, PA, Brazil; josygarcias19@gmail.com (J.T.G.); marceli.farias@icb.ufpa.br (M.R.D.O.F.); roseane.reis18@gmail.com (R.d.S.d.R.d.S.); dipaulamadeira@gmail.com (L.D.P.d.S.M.); herverogez@gmail.com (H.R.); jomontezanomarques@gmail.com (J.M.M.); 2 Faculty of Biology, Socioenvironmental and Water Resources Institute, Federal Rural University of the Amazon, Belém 66077-830, PA, Brazil; allana.lima.bl1@gmail.com (A.L.A.L.); hellen.kempfer@ufra.edu.br (H.K.P.)

**Keywords:** PGPR, *Bacillus proteolyticus*, *Priestia aryabhattai*, bioinoculant, *Euterpe oleracea*, drought tolerance

## Abstract

**Simple Summary:**

*Euterpe oleracea* Mart. is a palm tree native to the Amazon region, commonly found in humid areas such as floodplains. With the increasing demand for its fruit, the cultivation of this palm has expanded to upland areas, requiring high investments in irrigation. This study assessed the ability of bacteria isolated from the rhizosphere of açaí palms from both floodplain and upland areas, during dry and rainy seasons, to promote plant growth, especially under drought conditions. In total, 177 rhizobacteria were isolated. Among these were strains capable of producing the phytohormone indole acetic acid, synthesizing the enzyme ACC deaminase, solubilizing and mineralizing phosphates, and producing siderophores, among other characteristics. The majority of isolated strains (88%) inhibited the growth of phytopathogenic fungi *Curvularia* and *Colletotrichum*. Two strains, *Bacillus proteolyticus* and *Priestia aryabhattai*, were inoculated into açaí seeds and increased the speed and percentage of germination under conditions with either sufficient water supply or induced drought. Thus, these strains showed potential for use as biofertilizers and could contribute to sustainable agricultural practices.

**Abstract:**

*Euterpe oleracea* Mart., also known for its fruit açaí, is a palm native to the Amazon region. The state of Pará, Brazil, accounts for over 90% of açaí production. Demand for the fruit in national and international markets is increasing; however, climate change and diseases such as anthracnose, caused by the fungus *Colletotrichum* sp., lead to decreased production. To meet demand, measures such as expanding cultivation in upland areas are often adopted, requiring substantial economic investments, particularly in irrigation. Therefore, the aim of this study was to evaluate the potential of açaí rhizobacteria in promoting plant growth (PGPR). Rhizospheric soil samples from floodplain and upland açaí plantations were collected during rainy and dry seasons. Bacterial strains were isolated using the serial dilution method, and subsequent assays evaluated their ability to promote plant growth. Soil analyses indicated that the sampling period influenced the physicochemical properties of both areas, with increases observed during winter for most soil components like organic matter and C/N ratio. A total of 177 bacterial strains were isolated from rhizospheres of açaí trees cultivated in floodplain and upland areas across dry and rainy seasons. Among these strains, 24% produced IAA, 18% synthesized ACC deaminase, 11% mineralized organic phosphate, and 9% solubilized inorganic phosphate, among other characteristics. Interestingly, 88% inhibited the growth of phytopathogenic fungi of the genera *Curvularia* and *Colletotrichum*. Analysis under simulated water stress using Polyethylene Glycol 6000 revealed that 23% of the strains exhibited tolerance. Two strains were identified as *Bacillus proteolyticus* (PP218346) and *Priestia aryabhattai* (PP218347). Inoculation with these strains increased the speed and percentage of açaí seed germination. When inoculated in consortium, 85% of seeds germinated under severe stress, compared to only 10% in the control treatment. Therefore, these bacteria show potential for use as biofertilizers, enhancing the initial development of açaí plants and contributing to sustainable agricultural practices.

## 1. Introduction

*Euterpe oleracea* Mart, or açaí palm, is a native palm tree of the Amazon region, known for its abundance and production, with its main marketable product being the pulp of the fruit and its by-products [1]. It mainly grows in humid areas, being naturally adapted to environments with abundant water [2]. The roots of açaí are relatively shallow, meaning the plant relies on water near to the soil surface to absorb nutrients. This makes it sensitive to dry periods, which can negatively affect its growth and fruit production [1,2].

The state of Pará, in northern Brazil, is the world leader in açaí production. In 2022, the state’s production reached over 1.500 tons, representing more than 90% of the country’s total production of the fruit [3]. The plantation of palm trees in floodplain areas is well established, due to the tidal floods that increase water availability and soil fertility [4]. However, due to the growing demand for the fruit, there has been expansion into upland areas, where productivity can be affected by water deficit, reducing its physiological activities [1]. Thus, the increase in planted area in uplands also requires significant investments in irrigation systems, genetic improvement, and fertilization, making it a more viable venture for farmers with greater financial capacity [5].

Considering climate change and, in particular, the effects of El Niño in the Amazon, there are also environmental and public health concerns associated with the use of chemical fertilizers [6]. It is crucial to consider the impact of these practices, especially regarding the adaptation of açaí plants, given the importance of cultivation strategies to make them more resilient [5]. Additionally, it is essential to remember Brazil’s commitment to the UN’s 2030 agenda, which aims, through the Sustainable Development Goals (SDGs), to address issues such as mitigating climate change and promoting sustainable agricultural practices [7].

Drought is one of the major limiting factors for plant growth, negatively impacting agricultural productivity by reducing the availability of essential water for crucial physiological processes. The introduction of plant growth-promoting rhizobacteria (PGPRs) has proven to be an effective strategy to mitigate the adverse effects of drought. PGPRs, as described by Noureen, Iqbal, and Muqeet [8], act through various mechanisms including the production of indole-3-acetic acid (IAA), phosphate solubilization, ACC deaminase synthesis, and the production of siderophores and antimicrobial substances. These mechanisms promote root growth, improve nutrient absorption, and enhance resistance to water stress, providing plants with greater capability to survive and thrive under conditions of low water availability [7,8].

Based on this context, the search for more economically viable and sustainable alternatives becomes essential. Plant roots play a crucial role in recruiting PGPRs. Through the exudation of substances, roots attract microorganisms that, when associated with this structure, establish advantageous relationships for both parties [9]. Although the potential of PGPRs is already well elucidated, there are still few studies that address their specific interaction with and direct benefits for açaí palm trees, especially in environments with low water availability. Castro et al. [10,11] highlight some strains isolated from the rhizosphere of açaí palm trees and rice with the ability to strengthen the defense of the palm trees against phytopathogenic fungi and mitigate the effects of water deficit. Thus, further investigation of the bacterial communities in the rhizosphere of açaí palm trees in different environments, such as floodplains and solid ground, proves to be a potential alternative for the sustainable development of the açaí production chain.

We hypothesize that the açaí palm itself recruits beneficial bacteria in its rhizosphere, which can aid in plant growth even under biotic or abiotic stress conditions. Thus, in the present study, the potential for promoting plant growth of bacterial strains isolated from the rhizosphere of *E. oleracea* Mart. cultivated in floodplain and upland areas during dry and rainy seasons was evaluated. The practical application of the data obtained in this study can contribute to the promotion of sustainable agriculture in the Amazon region, helping to prevent the impacts of climate change and increase the resilience of trees to these changes.

## 2. Materials and Methods

### 2.1. Study Area

**Figure 1:**
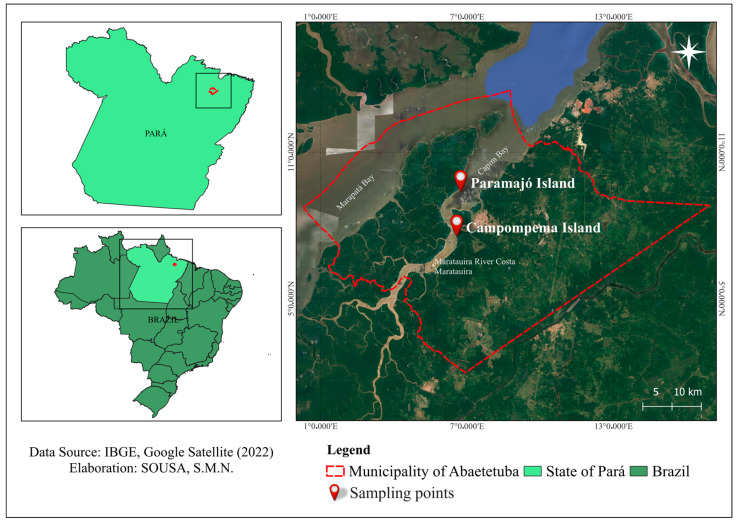
Location map of the sampling areas, Paramajó Island and Campompema Island, in the municipality of Abaetetuba, Pará, Brazil.

**Figure 2:**
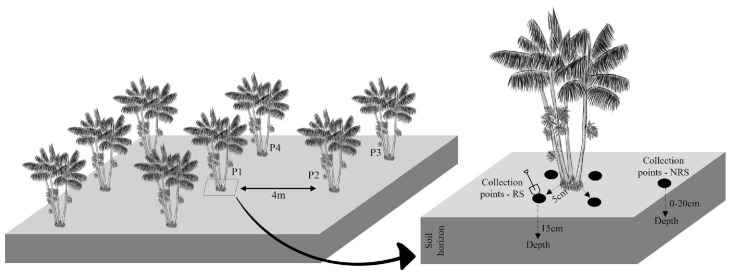
Experimental design exemplifying soil sampling planning. Legend: RS—rhizospheric soil; NRS—non-rhizospheric soil; P1—Plant 1; P2—Plant 2; P3—Plant 3; P4—Plant 4.

This study was conducted on two islands in Abaetetuba, Northeastern Mesoregion of Pará, Brazil, Paramajó (1°41′2.04″ S; 48°56′0.636″ W) and Campompema (1°44′39.8″ S; 48°55′09.5″ W) (Figure 1), with floodplain and upland açaí cultivations, respectively. Samples were collected in August 2021 (dry season) and March 2022 (rainy season). The physicochemical properties of the soils in the two areas are shown in Table 1, and the experimental design and biological material collection are detailed in Figure 2. In summary, rhizospheric soils were collected from four clumps of açaí trees, 5 cm off the stem, and within the first 15 cm of the soil horizon, totaling 16 composite samples in both periods and sampling areas. For physicochemical analyses, collections were made in the arable layer (0–20 cm deep) according to Embrapa instructions [12].

### 2.2. Isolation of Rhizobacteria

The isolation of bacterial strains was performed using the serial dilution method as described in the work of Sousa et al. [13]. Each dilution was plated in triplicate on Soybean Trypticase Agar (TSA) supplemented with 0.2% of the antifungal drug nystatin (0.1 mg/mL), and plates were incubated aerobically at 32 °C for up to 48 h. Taking into account the flooding conditions in the floodplain areas, which can also provide an environment for the survival of anaerobic and facultative bacteria, samples from these areas were also cultured under anaerobic conditions at the same temperature and time as the others, using anaerobic jars with BD GasPak™ sachets as indicators. Colony-forming units (CFUs) were counted, and Tukey’s test (*p* < 0.05) was applied for statistical analysis. Cell morphology was determined by Gram staining. All isolated strains were cryopreserved in triplicate in Tryptic Soy Broth (TSB) with 20% glycerol at −80 °C. The strains were named as follows: the letter A indicates the collection municipality (Abaetetuba-Pará); P is followed by the number of the plant replica (1, 2, 3, or 4); T indicates upland; V indicates floodplain, followed by the season (V for summer, I for winter); and finally the isolate number. Strains isolated under anaerobic conditions received an “^A^” before their isolation number, for example, AP4VV^A^3.

### 2.3. Pre-Inoculum and Selection of PGPRs

To perform the assays described below, a pre-inoculum of the strains previously isolated and preserved in glycerol was prepared. Thus, 50 µL of the glycerol culture was transferred to tubes containing 5 mL of TSB. The tubes were then kept for 24 h at a temperature of 32 °C with agitation at 150 rpm for strains cultured under aerobic conditions, while the others were cultured in an anaerobic jar, as described in Section 2.2 [13].

#### 2.3.1. Test for Inorganic Phosphate Solubilization and Organic Phosphate Mineralization

Strains were evaluated for their ability to solubilize inorganic phosphate using NBRIP (National Botanical Research Institute’s Phosphate) medium supplemented with 0.025 g/L of bromophenol blue (BPB) and 1.2% agar to visualize pH changes caused by the bacteria. For the organic phosphate mineralization assay, calcium phytate agar medium was used, where a positive result was indicated by clear halos around the colonies [14]. The quantitative evaluation of the phosphate solubilization and mineralization capacity was performed using the following equation:

$$\left(SI\right)=\frac{Cd+Hd}{Cd}$$

where SI = solubilization index, Cd = colony diameter, and Hd = halo diameter.

#### 2.3.2. Test for Siderophore Production

For the siderophore production test [15], 60.5 mg of chrome azurol S (CAS) was dissolved in 50 mL of distilled water and mixed with 10 mL of iron solution (FeCl_3.6_H_2_O 1 mM in HCl 10 mM). The mixture was added under agitation to a solution containing 72.9 mg of HDTMA in 40 mL of distilled water. The resulting solution was combined with iron-deficient KING B medium (at a ratio of 1:10) containing 1.2% agar and autoclaved, resulting in a blue color. Plates were inoculated with 5 µL of pre-inoculum (Section 2.3) and incubated at 28 °C for four to seven days. Siderophore production was indicated by a color change from blue to yellow. For quantitative estimation, bacteria positive in the qualitative test were incubated in KING B broth with agitation for 48 h. An aliquot of 1.5 mL was centrifuged for five minutes at 10,000 rpm. The supernatant was mixed with CAS solution, and after 30 min, absorbance was read at 630 nm. The assays were conducted in triplicate and analyzed by analysis of variance. Each sample was evaluated in terms of percentage of siderophore units (%SU) using the following formula:

$$\%SU=\frac{Ra-Sa}{Ra}\times 100$$

where Ra = reference absorbance (CAS reagent) and As = sample absorbance.

#### 2.3.3. Indole-3-Acetic Acid (IAA) Production Test

For the indole-3-acetic acid (IAA) production test [16], 50 µL of pre-inoculum culture was inoculated into 5 mL of KING B broth, maintained at 27 °C in the dark with agitation at 100 rpm. After 72 h, 1.5 mL of the cell suspension was centrifuged at 13,000 rpm for 10 min. The supernatant was mixed with Salkowski’s reagent, and after 30 min, a color change to pink indicated IAA production. For quantitative data collection, the IAA content produced by the strains was estimated by comparison with a standard curve calculated with known concentrations of the phytohormone (Figure S1).

#### 2.3.4. Biofilm Production

To assess biofilm production capacity, starting from the pre-inoculum (Section 2.3), each bacterium was streaked out with a sterile bacterial loop onto the surface of solid Congo Red agar medium, as described by Mendonça [17]. Plates were then incubated at 36 °C for 24 h, followed by further incubation at room temperature for 18 h. Congo Red is used as a pH indicator, where a change in the medium color to black demonstrates biofilm production capacity.

#### 2.3.5. Cellulolytic Activity

For the assessment of cellulolytic activity, the methodology described by Aguiar [18] was employed. Five microliters of pre-inoculum (Section 2.3) from each strain was applied onto JNFB agar medium containing 100 mg of yeast extract and 5 g of cellulose. Plates were then incubated at 30 °C for 4 days. After this period, 10 mL of Congo Red dye solution (2.5 g·L^−1^) in 0.1 M Tris HCl buffer, pH 8, was added. After 30 min, the solution was discarded, and the cultures were washed with 5 mL of 0.5 M NaCl. The observation of a halo around the colonies indicates cellulase enzyme production. The diameters of the halos were measured using a caliper to determine the enzymatic index (EI), which was calculated using the following equation:

$$EI=\frac{Cd+Hd}{Cd}$$

where EI = enzymatic index, Cd = colony diameter, and Hd = halo diameter.

#### 2.3.6. ACC Deaminase Enzyme Synthesis

To analyze the synthesis of the ACC deaminase, approximately 5 µL of each isolate cultured in TSB was inoculated onto plates containing ACC as the sole nitrogen source [0.1% K_2_HPO_4_; 0.02% MgSO_4.7_H_2_O; 0.01% FeSO_4.7_H_2_O; 0.1% CaCO_3_; 0.02% NaCl; 0.0005% Na_2_MoO_4.2_H_2_O; 1% glucose; 0.03% ACC (added by filtration); 1.5% agar]. The plates were incubated at 28 °C and observed daily for colony formation for up to 4 days. Colonies that showed growth after the incubation period were selected for subculturing on agar containing ACC as the sole nitrogen source, for confirmation of the test results [19].

#### 2.3.7. Test for Production of Antimicrobial Substances (AMS)

The production of antimicrobial substances was evaluated using the direct pairing method [20]. Bacterial strains were inoculated alongside phytopathogenic fungi *Curvularia* sp. (donated by Dr. Gisele Barata, from the Plant Protection Laboratory at the Federal Rural University of the Amazon) and *Colletotrichum* sp. (donated by Dr. Alberdan Santos, from the Systematic Investigation Laboratory in Biotechnology and Molecular Biodiversity at the Federal University of Pará). The indicator cultures were preserved on agar disks with glycerol solution (5%) and inoculated onto TSA. Bacterial strains were subcultured from the pre-inoculum (Section 2.3) using a sterile bacterial loop. Plates with both cultures were then incubated for seven days at 28 °C. A plate with only indicator cultures was used as a control for comparison.

### 2.4. Statistical Analyses

Quantitative analyses for the phosphate, siderophore, IAA, and cellulolytic activity assays were conducted for strains that showed positive results in at least three of the above-described PGPR assays. The data obtained were subjected to analysis of variance (ANOVA). The means were compared using Tukey’s test (*p* < 0.05). The analyses were performed using RStudio software, version 4.2.0.

### 2.5. Selection of Drought-Tolerant Strains

The bacterial strains that showed positive results in at least three of the assays mentioned earlier (Section 2.3) were evaluated for their tolerance to simulated drought with PEG 6000 [21]. The growth of cultures in TSB supplemented with different concentrations of PEG 6000 (−0.50, −0.75, and −1.0 Megapascal (MPa)) was measured using a spectrophotometer (UV-VISKASUAKI, IL—592) at 600 nm, with sterile TSB medium used as a blank. Measurements were performed in triplicate, and the optical density (OD) values were used to characterize the isolates as highly sensitive (OD < 0.3), sensitive (OD 0.3 to 0.39), tolerant (OD 0.4 to 0.5), and highly tolerant (OD > 0.5).

### 2.6. DNA Extraction and Molecular Identification

Genomic DNA of the strains selected for the next assays was extracted using the phenol–chloroform method [22], followed by amplification of the 16S rRNA gene via PCR. The PCR reaction used 1.0 µM of each universal primer 8F (5′AGAGTTTGATCATGGCTCAG3′) and 1492R (5′CGGTTACCTTGTTACGACTT3′), Promega^®^ 5× Green GoTaq^®^ Flexi Buffer at a concentration of 1×, 2 mM of MgCl_2_, 0.25 mM of dNTPs, 1 µL of GoTaq^®^ DNA polymerase, 1 µL (20 to 100 ng) of DNA, and sterile filtered water (Sigma-Aldrich^®^, Darmstadt, Germany) q.s.q. 50 µL. The applied cycle was 1× (5 min at 95 °C); 35× (1 min at 95 °C; 1 min at 55 °C; 1 min at 72 °C); 1× (10 min at 72 °C); 4 °C, using a GeneAmp PCR System 9700 thermocycler (Applied Biosystems) [23]. PCR products were purified using the PureLink^®^ PCR Purification Kit (Invitrogen™, Waltham, MA, USA) and sequenced using the Sanger method, performed on the Applied Biosystems 3500 Series genetic analyzer (Applied Biosystems, Waltham, MA, USA^®^). The approximately 1500 bp sequences were analyzed using the BioEdit program version 7.2.5. [24] and compared with sequences in GenBank using the NCBI’s nucleotide BLAST tool (BLASTn). Similar type strain sequences were obtained from NCBI, and a phylogenetic tree was constructed using MEGA 11 software [25] employing the Neighbor-Joining method with 1000 bootstrap replicates. The 16S rRNA sequences obtained in this study were also deposited in NCBI (GenBank: PP218346 and PP218347).

### 2.7. Effect of PGPR Inoculation on Seed Germination and Initial Seedling Development of Açaí under Water Deficiency

The seeds of native açai from the northeast mesoregion of Pará were subjected to germination tests with and without inoculation of rhizobacteria, both individually and in combination. Different concentrations of PEG 6000 were applied to create osmotic potential gradients (0 MPa, −0.50 MPa, and −1.0 MPa) [26]. Before testing, the seeds were sanitized with 70% ethanol and sodium hypochlorite (2.5%), rinsed with distilled water, and treated with a nystatin fungicide solution (0.2%), followed by several rinses with sterile distilled water. For microbiolization, the seeds were immersed in bacterial suspensions in phosphate-buffered saline (PBS) with optical density adjusted to 0.6 (~10^8^ CFU/mL) and kept overnight at room temperature with agitation at 150 rpm. Control seeds were treated only with sterile PBS. After microbiolization, the seeds were transferred to Petri dishes containing a layer of sterile hydrophilic cotton soaked with 10 mL of PEG 6000, simulating the osmotic potentials described above. Each treatment had four replicates with 15 seeds each, totaling 60 seeds per treatment. The plates were kept in a growth chamber at 28 °C, under a 12 h photoperiod, and the germination index was monitored daily. After germination stabilization, the seedlings were carefully removed, and length measurements were taken for both shoots and roots. The percentage of germination and vigor index were calculated as described by Chukwuneme et al. [27]:

Germination rate (%) = nN × 100

where n is the number of germinated seeds and N is the total number of seeds.

Vigor index = % Germination × total length of seedling.

Statistical analyses were performed as described in Section 2.3. It is worth noting that to ensure the selected bacteria were not antagonistic to each other, a direct pairing test was conducted with the strains [20].

## 3. Results

### 3.1. Isolation, Selection of PGPRs, and Evaluation of Drought Tolerance

The average counts of rhizobacteria in CFU in upland plants during both dry and rainy periods were on average 9 × 10^3^ CFU/g of soil. In the floodplain area, the average CFU counts during the dry season were 8 × 10^3^ CFU/g under both anaerobic and aerobic conditions, while during the rainy season, the averages were 2 × 10^4^ CFU/g under anaerobic conditions and 1 × 10^4^ CFU/g under aerobic conditions (*p* > 0.05).

In total, 177 bacterial strains were isolated. Collection during the dry season resulted in the isolation of 93 strains, with 32 from upland areas and 61 from floodplains, while collection during the rainy season resulted in the isolation of 84 strains, 19 from upland areas and 65 from floodplains. 

Among all isolated strains (Table S1), 88% produced antimicrobial substances, with 85% inhibiting the growth of the phytopathogenic fungus *Curvularia* sp., 51% inhibiting the growth of *Colletotrichum* sp., and 49% inhibiting the growth of both fungi. Additionally, 24% were capable of producing IAA, 18% were capable of synthesizing ACC deaminase, 12% formed biofilms, 11% solubilized inorganic phosphate, 10% showed cellulolytic capacity, 9% mineralized organic phosphate, and 8% produced siderophores. Only 12 strains did not show positive results for any of the analyses performed. In contrast, 22 were positive for at least three of the assays conducted (Table 2).

The 22 strains that showed the best results for PGPR assays were evaluated for their tolerance to water deficiency induced with PEG 6000. The results showed that five of the strains were classified as highly sensitive, seven as sensitive, five as tolerant, and another five as highly tolerant (Table 2).

### 3.2. Molecular Identification and Phylogenetic Analysis

**Figure 3:**
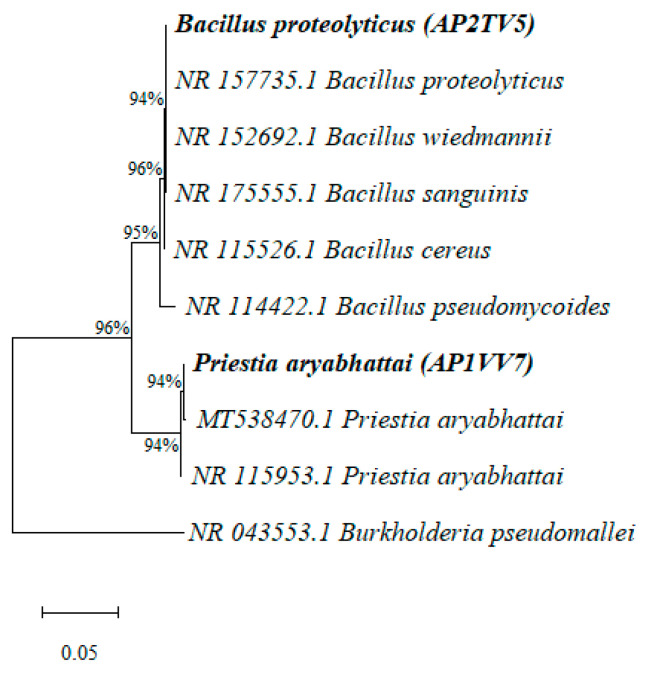
Phylogenetic analysis of the *B. proteolyticus* (AP2TV5) and *P. aryabhattai* (AP1VV7) strains based on 16S rRNA sequences. The Neighbor-Joining method was used in MEGA 11, with bootstrap values (n = 1000) indicated on branches.

Two strains, AP2TV5 and AP1VV7, were selected based on criteria such as being Gram-positive, producing IAA, synthesizing the enzyme ACC deaminase, and being tolerant to drought. These strains were identified as *Bacillus proteolyticus* and *Priestia aryabhattai*, respectively. Both showed 100% similarity with reference sequences in GenBank. Phylogenetic analysis confirmed the genetic relationship between these isolates, placing them in the same clades as the closest sequences from the database, *B. proteolyticus* (NR_157735.1) and *P. aryabhattai* (NR_115953.1). Furthermore, the clades in which the two strains are located also share a close evolutionary relationship (96%) (Figure 3).

### 3.3. Assessment of Germination Percentage and Speed Index

**Figure 4:**
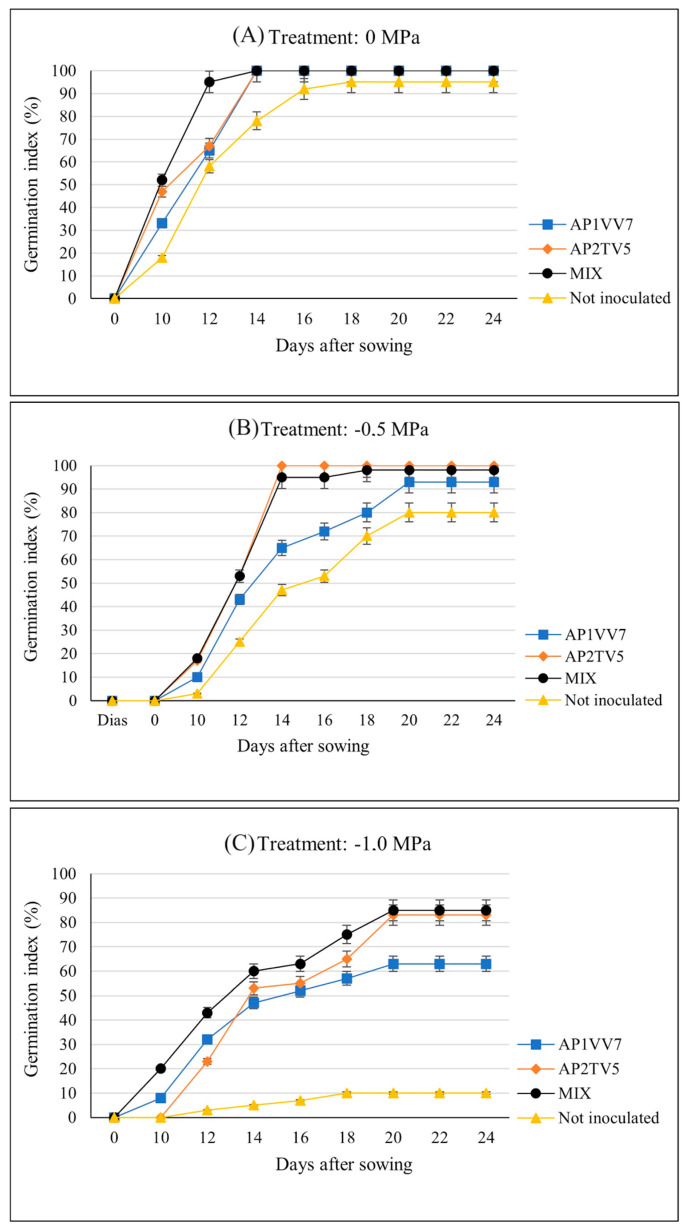
Germination index and speed of germination of seeds of *E. oleracea* Mart. non-inoculated and inoculated with strains *B. proteolyticus* (AP2TV5) and *P. aryabhattai* (AP1VV7), separately and in association, and subjected to different treatments with and without addition of PEG 6000: (**A**) without addition of PEG 6000 (0 MPa), (**B**) with addition of PEG 6000 (−0.5 MPa), and (**C**) with addition of PEG 6000 (−1.0 MPa). Each treatment had four repetitions with 15 seeds each, totaling 60 seeds per treatment.

The inoculation of *B. proteolyticus* (AP2TV5) and *P. aryabhattai* (AP1VV7) strains, either individually or in consortium, increased the germination of açaí seeds both under water deficiency conditions and normal conditions. All inoculated treatments achieved 100% germination under normal conditions, while the control reached 95%. Under moderate stress (−0.5 MPa), *B. proteolyticus* performed better, and under more severe stress (−1.0 MPa), all inoculated treatments significantly outperformed the control (Table 3). Regarding germination speed, under non-stress conditions, it stabilized within 14 days, while the control took 18 days. Under moderate stress, both the consortium and *B. proteolyticus* reached maximum germination in 18 days, and under severe stress, the control stabilized in 18 days but with only 10% germination, while the others reached maximum germination in 20 days. Germination was observed up to 24 days after sowing (Figure 4).

### 3.4. Initial Growth of Açaí Seedlings

**Figure 5:**
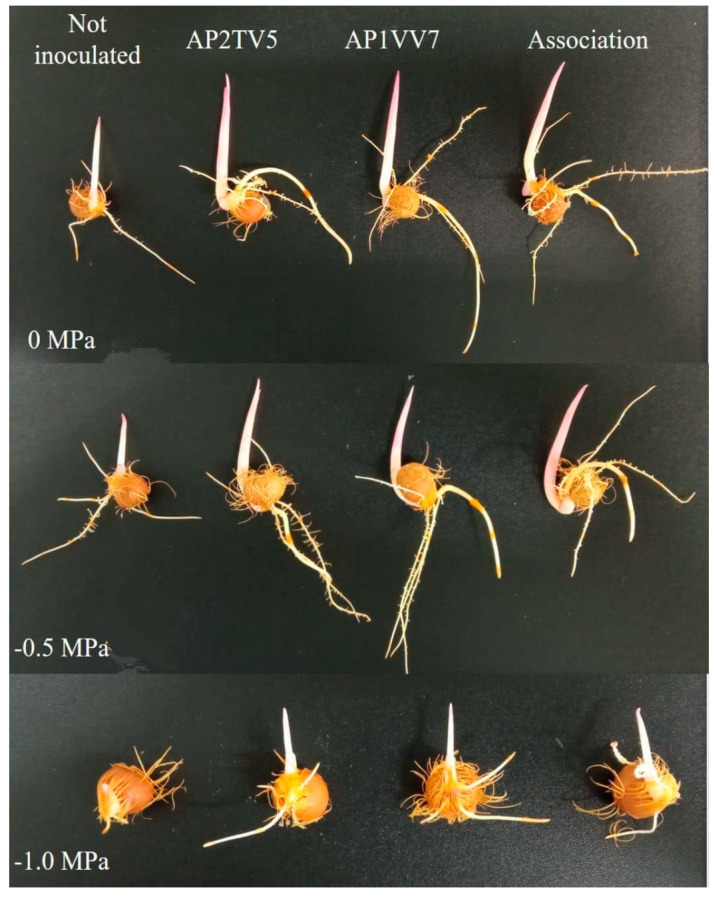
Initial development of açai seedlings from non-inoculated and inoculated seeds with PGPRs subjected to different treatments with and without the addition of PEG 6000 to simulate water stress. Seeds aligned from top to bottom were subjected to treatments at 0, −0.5, and −1.0 MPa, and from left to right columns: non-inoculated, inoculated with *B. proteolyticus* (AP2TV5) strain, inoculated with *P. aryabhattai* (AP1VV7) strain, and inoculated with both strains in association.

Seeds inoculated with PGPRs showed an interesting potential for the initial development of plants (Table 4). Regarding root length, under non-stress conditions, there was no statistically significant difference. However, when subjected to water deficiency of −0.5 MPa and −1.0 MPa, all inoculated treatments exhibited significantly greater growth compared to the control, with inoculation of the strain *P. aryabhattai* (AP1VV7) inducing the greatest root growth (Figure 5). As for the number of roots, there was no significant difference, with the number ranging between three and five per seed. Regarding initial seedling length and seed vigor index, seeds inoculated with PGPRs showed superior results compared to the control. There was no statistically significant difference among the inoculated treatments. The seedlings were observed until day 45 after inoculation.

## 4. Discussion

The rhizospheric soils of açaí palm trees grown in floodplain and upland areas harbor various bacterial strains with the potential to promote plant growth, even under conditions of biotic and abiotic stresses. In this study, according to the presented hypothesis, the majority of isolated strains demonstrated functional capabilities that can directly or indirectly influence plant development, especially the ability to produce antimicrobial substances against phytopathogenic fungi, to produce IAA (indole-3-acetic acid), and to synthesize the enzyme ACC (1-aminocyclopropane-1-carboxylate) deaminase. 

The production of the phytohormone IAA promotes root development, increasing nutrient uptake by plants [28,29]. The production of antimicrobial substances, in turn, shows potential in controlling phytopathogens [30,31], such as the fungi *Curvularia* sp. and *Colletotrichum* sp., already isolated from açaí plants [32], which can cause up to 70% loss of seedlings in nurseries [11]. The ability of strains to synthesize ACC deaminase can help plants tolerate stress caused by ethylene accumulation, especially under abiotic stresses such as water deficiency [33,34].

To the best of our knowledge, this is the first study to simultaneously analyze the cultivable bacterial community of açai plantations from different areas, both in the dry and rainy seasons. Although more strains were isolated from the floodplain environment, rhizobacteria that showed positive results in at least three PGPR assays were more prevalent in upland areas during the dry season, which coincides with the açai harvest. A combination of environmental factors [35,36] and microbial adaptations could explain this predominance. The specific soil conditions (organic matter, C/N, pH, among others; Table 1) and climate in this environment appear to be more favorable to these strains. Furthermore, açai plants in upland areas may be more predisposed to form effective symbioses with these rhizobacteria, developing adaptive strategies to thrive in this environment and making them more competitive during the Amazonian summer.

Precise identification of the strains is also essential to understand their ecology, physiology, and potential application in different contexts, such as agriculture. Here, the strains AP2TV5 and AP1VV7 were identified as *Bacillus proteolyticus* and *Priestia aryabhattai*, respectively. Other studies have already demonstrated the biotechnological potential of these species. Meza et al. [37] identified *B. proteolyticus* (Cyn 1) as a plant growth promoter, supporting the germination and growth of *Phaseolus vulgaris* L. under abiotic stress conditions such as temperature, water, and salinity stress. Like the strain AP2TV5 isolated here, the strain Cyn 1 was also identified as capable of producing ACC deaminase, facilitating the activity of these strains in favor of plant growth. In addition to its plant-promoting activity, *B. proteolyticus* has also been cited as a species with potential for remediating soils contaminated with heavy metals [38] and even to possess probiotic properties [39].

*P. aryabhattai*, also referred to as *Bacillus aryabhattai*, has shown potential to enhance growth and nutrient absorption in bean and maize crops when inoculated in consortium with *B. subtilis* [40], as well as in rice cultivation, displaying high IAA production capacity [41]. Moreover, interestingly, *B. aryabhattai* KNUC205, isolated from an urban tunnel, exhibited antifungal activity, demonstrated crack remediation, and reduced water permeability in cement mortar pastes [42].

In the current study, *B. proteolyticus* (AP2TV5) and *P. aryabhattai* (AP1VV7) were able to increase the percentage and speed of germination of açaí seeds under both drought and normal conditions (Table 3). The fact that inoculated seeds reached germination stabilization faster than the control treatment under all evaluated conditions highlights the soil bacteria’s ability to accelerate the germination process, corroborating the results found in in vitro assays of PGPR potential. Our results are in line with several studies that also report the potential of rhizobacteria to increase the germination index and speed of seeds from various plant species under water deficiency conditions [11,21,43,44].

Evaluation of root growth parameters and seedling length also revealed that all PGPR-inoculated treatments had positive effects on the initial development of plants. Similar data were obtained by Ahmed et al. [45] when inoculating a drought-tolerant PGPR strain (*Enterobacter* sp./*Leclercia adecarboxylata* PAB19) in *Vigna radiata* (mung bean), resulting in a significant increase in root growth. Here, specifically, inoculation with the *P. aryabhattai* (AP1VV7) strain proved to be the most effective in promoting root growth under severe water deficiency conditions (−1.0 MPa), which may be related to the high potential of this strain to produce IAA.

Regarding the initial length of seedlings and the seed vigor index of açai, all treatments with PGPR inoculation showed results indicating a positive effect of inoculation on seedling vigor and initial development, which corroborates the findings of Castro et al. [10], who evaluated PGPRs isolated from the rhizosphere of rice and açai. Thus, both the isolated inoculation of strains and the co-inoculation of them brought satisfactory results for promoting seedling growth. Wang et al. [46] also reported that co-inoculation of PGPRs can increase plant tolerance to abiotic stresses. This approach has significant implications for the sustainable management of açaí plantations, offering a viable alternative to increase plant resilience and improve productivity in challenging environments.

## 5. Conclusions

Given the results obtained in this research, it is evident that the rhizospheric soils of açaí palms are rich in bacterial strains with distinct functional capabilities that can significantly influence plant growth and development. These capabilities include phosphate solubilization and mineralization, IAA production, ACC deaminase synthesis, and the production of antimicrobial substances against phytopathogenic fungi, among other characteristics. Additionally, the identification of strains with plant growth-promoting activities highlights their potential to boost açaí plantation productivity, especially during drought periods. The selection and identification of *B. proteolyticus* and *P. aryabhattai* strains as plant growth promoters with drought tolerance capabilities provide a solid foundation for future agricultural applications. These strains have shown promising results in enhancing germination and initial seedling development of açaí, even under severe water deficiency conditions. Therefore, these findings are of great practical importance and play a crucial role in future research aimed at improving the productivity and sustainability of açaí plantations in the Amazon region, especially in the face of climate change and the increasing demand for more sustainable agricultural practices.

## Figures and Tables

**Table 1:** Physicochemical properties of soils from floodplain and upland areas with *E. oleracea* Mart. cultivation in the municipality of Abaetetuba during the summer and winter periods.

Area	Depth	C	MO	N	N	Ratio	P	K	Na	Al	Ca	Ca+Mg	pH
	(cm)	* g/kg		%	* g/kg	C/N	** mg/dm^3^			*** cmolc/dm^3^			H_2_O
**Summer**													
Floodplain soil	20	6.84	11.8	0.18	1.8	3.81	5	47	40	0.13	7.1	10.13	6.01
Solid ground	20	12.5	21.59	0.04	0.36	34.71	2	14	7	1.7	0.2	0.37	5.24
**Winter**													
Floodplain soil	20	14.8	25.46	0.03	0.31	47.79	9.59	65.3	63.7	0.11	5.7	9.54	5.18
Solid ground	20	15.3	26.46	0.01	0.1	153.4	10.47	22.7	19.7	1.6	0.2	0.8	4.78

* g/kg: quantity in grams per kilogram of soil; ** mg/dm^3^: quantity in milligrams per cubic decimeter of soil; *** cmolc/dm^3^: centimoles of nutrient charge per cubic decimeter of soil.

**Table 2:** Characteristics of rhizobacteria isolated from *E. oleracea* Mart. that showed positive results for at least three of the analyses conducted to assess their potential for promoting plant growth.

Strain	Sampling Area	Season	Cell Morphology (Gram)	IAA (µg/mL)	Organic Phosphate (MI)	Inorganic Phosphate (SI)	Siderophores (%US)	Cellulolytic Activity (EI)	Biofilm	ACC Deaminase	AMS Production		Water Stress Tolerance
											*Curvularia*	*Colletotrichum*	
AP1TV1	Solid ground	Summer	Streptobacilli (+)	-	14.04 bc	-	-	12.58 a	-	+	+	+	Highly sensitive
AP1TV5	Solid ground	Summer	Streptobacilli (+)	-	-	7.67 c	-	7.10 d	-	+	+	+	Highly tolerant
AP1TV7	Solid ground	Summer	Streptobacilli (+)	-	12.07 cde	-	-	7.08 d	-	+	+	+	Sensitive
AP2TV1	Solid ground	Summer	Streptobacilli (+)	-	19.24 a	-	-	8.44 cd	-	+	+	-	Sensitive
AP2TV4	Solid ground	Summer	Diplobacilli (+)	-	12.42 cde	7.38 c	-	-	-	+	+	+	Tolerant
AP2TV5	Solid ground	Summer	Diplobacilli (+)	2.70 ab	-	7.52 c	-	8.89 bc	-	+	+	+	Tolerant
AP2TV8	Solid ground	Summer	Bacilli (+)	0.98 c	-	-	96.56 a	7.73 cd	-	+	+	+	Sensitive
AP3TV2	Solid ground	Summer	Bacilli (+)	-	-	-	-	7.78 cd	+	+	+	+	Highly tolerant
AP3TV5	Solid ground	Summer	Bacilli (+)	-	16.62 ab	-	-	7.10 d	-	-	+	+	Sensitive
AP4TV1	Solid ground	Summer	Diplobacilli (+)	2.09 bc	-	-	96.56 a	-	-	-	+	+	Sensitive
AP4TV3	Solid ground	Summer	Bacilli (+)	1.21 c	-	-	-	9.92 b	-	+	+	+	Sensitive
AP4TV4	Solid ground	Summer	Bacilli (-)	0.92 c	7.81 f	-	-	-	-	-	+	-	Highly sensitive
AP1VV1	Floodplain	Summer	Streptobacilli (+)	1.92 bc	-	7.38 c	-	-	-	+	+	+	Highly tolerant
AP1VV7	Floodplain	Summer	Diplobacilli (+)	4.00 a	-	11.01 a	-	9.97 b	+	+	+	-	Tolerant
AP3VV1	Floodplain	Summer	Streptobacilli (+)	1.80 bc	-	-	-	-	+	+	+	-	Highly sensitive
AP3VV9	Floodplain	Summer	Streptobacilli (+)	-	11.67 cde	7.83 c	-	-	-	+	+	+	Tolerant
AP4VV4	Floodplain	Summer	Coccos (-)	1.24 c	12.83 cd	-	-	-	-	-	+	-	Highly sensitive
AP4VV5	Floodplain	Summer	Streptobacilli (+)	-	10.24 def	-	-	7.78 cd	-	-	+	-	Highly sensitive
AP4VV^A^3	Floodplain	Summer	Coccos (-)	1.55 bc	-	10.57 a	96.13 a	7.67 cd	-	+	-	-	Highly tolerant
AP2TI5	Solid ground	Winter	Coccobacillus (-)	-	10.07 def	10.11 ab	96.20 a	-	+	+	-	-	Tolerant
AP1VI3	Floodplain	Winter	Coccobacillus (-)	1.86 bc	9.28 ef	9.17 b	96.60 a	-	-	+	+	-	Highly tolerant
AP2VI7	Floodplain	Winter	Coccobacillus (-)	-	10.08 def	10.96 a	94.86 b	-	+	+	+	-	Sensitive

Caption: (+) positive result; (-) negative result; (SI) inorganic phosphate solubilization index; (MI) organic phosphate mineralization index; (%SU) siderophore unit; (EI) enzymatic index. Values followed by the same letters in the columns do not differ statistically from each other (*p* ≤ 0.05) in the Tukey test.

**Table 3:** Percentage of germination of non-inoculated and inoculated açai seeds with *B. proteolyticus* (AP2TV5) and *P. aryabhattai* (AP1VV7) strains subjected to simulated water stress conditions with PEG 6000. Values followed by the same letters (uppercase in columns and lowercase in rows) do not differ statistically (*p* < 0.05) in the Tukey test.

Treatment	Germination %		
	0 MPa	−0.5 MPa	−1.0 MPa
Not inoculated	95.00 ± 0.96 Aa	80.00 ± 2.16 Ba	10.00 ± 0.58 Bb
AP2TV5	100.00 ± 0.00 Aa	100.00 ± 0.00 Aa	83.00 ± 1.29 Ab
AP1VV7	100.00 ± 0.00 Aa	93.00 ± 0.81 ABa	63.00 ± 2.38 Ab
Association	100.00 ± 0.00 Aa	98.00 ± 0.50 Aab	85.00 ± 1.70 Ab

**Table 4:** Evaluation of root growth, length, and vigor index of *E. oleracea* Mart. seedlings non-inoculated and inoculated with strains *B. proteolyticus* (AP2TV5) and *P. aryabhattai* (AP1VV7), separately and in association, and subjected to simulated water stress conditions with PEG 6000. Values followed by the same letters within columns did not differ statistically (*p* < 0.05) in the Tukey test.

Treatment	Root Length (cm)			Seedling Length (cm)			Vigor Index		
	0 MPa	−0.5 MPa	−1.0 MPa	0 MPa	−0.5 MPa	−1.0 MPa	0 MPa	−0.5 MPa	−1.0 MPa
Not inoculated	2.92 ± 1.09 Aa	2.42 ± 0.98 Ba	0.50 ± 0.08 Cb	2.90 ± 0.17 Ba	2.03 ± 0.05 Ba	0.90 ± 0.10 Bb	275.5	162.4	9.0
AP2TV5	4.22 ± 1.78 Aa	4.15 ± 0.44 Aa	1.27 ± 0.48 Bb	5.33 ± 0.11 Aa	4.37 ± 0.15 Aa	2.17 ± 0.15 Ab	533.0	437.0	157.7
AP1VV7	4.22 ± 0.22 Aa	5.27 ± 1.05 Aa	2.12 ± 0.30 Ab	5.30 ± 0.26 Aa	4.17 ± 0.15 Aa	1.90 ± 0.10 Ab	530.0	387.8	136.7
Association	5.05 ± 0.90 Aa	4.70 ± 0.48 Aa	1.65 ± 0.24 Ab	5.10 ± 0.10 Aa	4.33 ± 0.15 Aa	2.20 ± 0.09 Ab	510.0	424.3	187.0

## Data Availability

Data are contained within the article and Supplementary Materials.

## Supplementary Materials

The following supporting information can be downloaded at: https://www.mdpi.com/article/10.3390/biology13100757/s1, Figure S1: Standard curve for determining the concentration of the phytohormone indole-3-acetic acid (Sigma-I3750); Table S1: General characteristics of rhizobacteria isolated from *E. oleracea* Mart. and evaluated for their potential to promote plant growth.
