# Supplementary material for: Rhizobacteria Isolated from Amazonian Soils Reduce the Effects of Water Stress on the Growth of Açaí (Euterpe oleracea Mart.) Palm Seedlings
Source: Biology (Basel). 2024 Sep 24;13(10):757. doi: 10.3390/biology13100757 (PMC11504209; doi:10.3390/biology13100757)
Supplement: Supplementary file 1 [file biology-13-00757-s001.zip › Table S1 - Copia.pdf]

Table S1: General characteristics of rhizobacteria isolated from *E. oleracea* Mart. and evaluated for their potential in promoting plant growth.

Strain Identification	Sampling Area	Season	Gram Staining	Cell Morphology	IAA	Organic phosphate	Inorganic phosphate	Siderophores	Cellulolytic activity	Biofilm	ACC Deaminase	AMS production	
												Curvularia	*Colletotrichum*
AP1TV1	Solid ground	Summer	+	Streptobacilli	-	+	-	-	+	-	+	+	+
AP1TV2	Solid ground	Summer	+	Streptococci	+	-	-	-	-	-	-	+	-
AP1TV3	Solid ground	Summer	+	Diplobacilli	-	-	-	-	+	-	-	-	+
AP1TV4	Solid ground	Summer	+	Coccos	+	-	-	-	-	-	-	+	+
AP1TV5	Solid ground	Summer	+	Streptobacilli	-	-	+	-	+	-	+	+	+
AP1TV6	Solid ground	Summer	+	Bacilli	+	-	-	-	-	-	-	+	+
AP1TV7	Solid ground	Summer	+	Streptobacilli	-	+	-	-	+	-	+	+	+
AP1TV8	Solid ground	Summer	+	Bacilli	+	-	+	-	-	-	-	+	+
AP1TV9	Solid ground	Summer	+	Bacilli	-	+	-	-	-	-	-	+	+
AP1TV10	Solid ground	Summer	+	Coccobacillus	-	-	-	-	-	-	+	+	+
AP1TV11	Solid ground	Summer	+	Bacilli	+	-	-	-	-	-	-	+	+
AP2TV1	Solid ground	Summer	+	Streptobacilli	-	+	-	-	+	-	+	+	-
AP2TV2	Solid ground	Summer	-	Staphylococci	-	-	-	-	-	-	+	+	-
AP2TV3	Solid ground	Summer	+	Bacilli	-	-	-	-	-	-	-	-	-
AP2TV4	Solid ground	Summer	+	Diplobacilli	-	+	+	-	-	-	+	+	+
AP2TV5	Solid ground	Summer	+	Diplobacilli	+	-	+	-	+	-	+	+	+
AP2TV6	Solid ground	Summer	+	Coccos	+	-	-	-	-	-	-	+	+
AP2TV7	Solid ground	Summer	+	Streptobacilli	-	-	-	-	-	-	+	+	+
AP2TV8	Solid ground	Summer	+	Bacilli	+	-	-	+	+	-	+	+	+
AP3TV1	Solid ground	Summer	-	Staphylococci	-	-	-	-	-	-	+	+	+
AP3TV2	Solid ground	Summer	+	Bacilli	-	-	-	-	+	+	+	+	+
AP3TV3	Solid ground	Summer	-	Bacilli	-	-	-	-	-	-	-	-	-
AP3TV5	Solid ground	Summer	+	Bacilli	-	+	-	-	+	-	-	+	+
AP3TV6	Solid ground	Summer	+	Bacilli	-	-	-	-	-	-	+	+	+
AP3TV7	Solid ground	Summer	+	Coccobacillus	+	-	-	-	-	-	-	+	+
AP3TV8	Solid ground	Summer	+	Coccobacillus	+	-	-	-	-	-	-	+	+

AP4TV1	Solid ground	Summer	+	Diplobacilli	+	-	-	+	-	-	-	+	+
AP4TV2	Solid ground	Summer	-	Staphylococci	-	-	-	+	-	-	-	+	-
AP4TV3	Solid ground	Summer	+	Bacilli	+	-	-	-	+	-	+	+	+
AP4TV4	Solid ground	Summer	-	Bacilli	+	+	-	-	-	-	-	+	-
AP4TV5	Solid ground	Summer	-	Bacilli	-	-	-	-	-	-	-	-	-
AP4TV7	Solid ground	Summer	+	Coccobacillus	-	-	-	-	-	+	-	+	-
AP1VV1	Floodplain	Summer	+	Staphylococci	+	-	+	-	-	-	+	+	+
AP1VV2	Floodplain	Summer	+	Diplobacilli	-	-	-	-	-	-	+	+	+
AP1VV3	Floodplain	Summer	+	Bacilli	+	-	-	-	-	-	-	+	+
AP1VV4	Floodplain	Summer	+	Bacilli	-	-	-	-	-	-	-	-	-
AP1VV5	Floodplain	Summer	-	Bacilli	-	-	+	-	-	-	-	+	+
AP1VV6	Floodplain	Summer	+	Bacilli	-	-	-	-	-	-	-	-	-
AP1VV7	Floodplain	Summer	+	Diplobacilli	+	-	+	-	+	+	+	+	-
AP1VV8	Floodplain	Summer	+	Coccobacillus	-	-	+	-	-	-	-	+	+
AP2VV1	Floodplain	Summer	+	Diplobacilli	+	-	-	-	-	-	-	+	-
AP2VV2	Floodplain	Summer	-	Coccobacillus	-	-	-	-	-	-	+	+	+
AP2VV3	Floodplain	Summer	+	Diplobacilli	-	-	-	-	-	-	-	-	-
AP2VV4	Floodplain	Summer	+	Diplobacilli	+	-	-	-	-	-	-	+	+
AP3VV1	Floodplain	Summer	+	Streptobacilli	+	-	-	-	-	+	+	+	-
AP3VV2	Floodplain	Summer	+	Streptobacilli	+	-	-	-	-	-	-	+	-
AP3VV3	Floodplain	Summer	-	Spirochete	-	-	-	-	-	+	-	+	-
AP3VV4	Floodplain	Summer	+	Diplobacilli	-	-	-	+	-	-	+	+	-
AP3VV5	Floodplain	Summer	-	Coccos	-	-	-	-	-	-	+	+	+
AP3VV6	Floodplain	Summer	+	Bacilli	-	-	-	-	-	-	+	+	+
AP3VV7	Floodplain	Summer	+	Diplobacilli	-	-	-	+	-	-	-	+	-
AP3VV8	Floodplain	Summer	+	Diplobacilli	-	-	-	-	-	-	+	+	-

AP3VV9	Floodplain	Summer	+	Vibrio	-	+	+	-	-	-	+	+	+
AP4VV1	Floodplain	Summer	+	Bacilli	+	-	-	-	-	-	-	+	-
AP4VV2	Floodplain	Summer	-	Bacilli	+	-	-	-	-	-	-	+	-
AP4VV3	Floodplain	Summer	+	Bacilli	-	+	-	-	-	-	-	+	+
AP4VV4	Floodplain	Summer	-	Coccos	+	+	-	-	-	-	-	+	-
AP4VV5	Floodplain	Summer	+	Streptobacilli	-	+	-	-	+	-	-	+	-
AP4VV6	Floodplain	Summer	+	Streptobacilli	-	-	-	+	-	-	-	+	-
AP4VV7	Floodplain	Summer	+	Bacilli	-	-	-	-	-	-	-	+	+
AP4VV8	Floodplain	Summer	+	Bacilli	-	+	-	-	-	-	-	+	+
AP1VV^{A}1	Floodplain	Summer	+	Bacilli	+	-	-	-	-	-	-	+	-
AP1VV^{A}2	Floodplain	Summer	-	Coccos	-	-	-	-	+	-	-	+	+
AP1VV^{A}3	Floodplain	Summer	+	Bacilli	-	-	-	-	-	-	-	+	-
AP1VV^{A}4	Floodplain	Summer	+	Diplobacilli	-	-	-	-	-	-	-	+	-
AP1VV^{A}5	Floodplain	Summer	+	Diplobacilli	-	-	-	-	-	-	-	+	-
AP1VV^{A}6	Floodplain	Summer	+	Diplobacilli	+	-	+	-	-	-	-	-	-
AP1VV^{A}7	Floodplain	Summer	+	Streptobacilli	-	-	-	-	+	-	-	+	+
AP1VV^{A}8	Floodplain	Summer	+	Diplobacilli	+	-	+	-	-	-	-	-	-
AP1VV^{A}9	Floodplain	Summer	+	Streptobacilli	-	-	-	-	-	-	-	-	-
AP1VV^{A}10	Floodplain	Summer	-	Coccos	-	-	+	-	+	-	-	-	+
AP2VV^{A}1	Floodplain	Summer	+	Bacilli	-	-	-	-	-	-	+	+	-
AP2VV^{A}2	Floodplain	Summer	-	Coccos	-	-	+	-	-	-	-	+	+
AP2VV^{A}3	Floodplain	Summer	-	Coccos	-	-	+	-	-	-	-	+	+
AP2VV^{A}4	Floodplain	Summer	+	Bacilli	-	-	-	-	-	-	+	+	-
AP2VV^{A}5	Floodplain	Summer	+	Coccos	-	-	-	+	-	-	-	-	-
AP2VV^{A}6	Floodplain	Summer	+	Coccos	-	-	-	-	-	-	-	+	-
AP2VV^{A}7	Floodplain	Summer	+	Streptobacilli	-	-	-	-	-	-	-	-	-

AP2VV^{A}8	Floodplain	Summer	+	Coccos	-	-	-	-	-	-	-	+	-
AP2VV^{A}9	Floodplain	Summer	+	Bacilli	-	-	-	-	-	-	-	+	-
AP3VV^{A}3	Floodplain	Summer	+	Bacilli	-	-	-	-	-	-	+	+	+
AP3VV^{A}4	Floodplain	Summer	+	Coccos	-	-	-	-	-	-	-	+	+
AP4VV^{A}1	Floodplain	Summer	+	Streptobacilli	-	-	-	-	-	+	-	-	-
AP4VV^{A}2	Floodplain	Summer	+	Bacilli	-	-	-	-	-	-	-	-	-
AP4VV^{A}3	Floodplain	Summer	-	Coccos	+	-	+	+	+	-	+	-	-
AP4VV^{A}4	Floodplain	Summer	-	Coccos	-	-	-	-	-	-	-	+	+
AP4VV^{A}5	Floodplain	Summer	+	Streptobacilli	-	-	-	-	-	+	-	-	-
AP4VV^{A}6	Floodplain	Summer	+	Streptobacilli	-	-	-	-	-	-	-	+	+
AP4VV^{A}7	Floodplain	Summer	+	Bacilli	+	-	-	-	-	-	+	-	-
AP4VV^{A}8	Floodplain	Summer	+	Bacilli	-	-	-	-	-	-	-	-	-
AP4VV^{A}9	Floodplain	Summer	-	Streptobacilli	-	-	-	-	-	-	-	-	-
AP4VV^{A}10	Floodplain	Summer	+	Diplobacilli	+	-	-	-	-	+	-	-	+
AP4VV^{A}11	Floodplain	Summer	-	Coccos	-	-	-	-	-	-	-	-	+
AP1TI1	Solid ground	Winter	+	Diplobacilli	-	-	-	-	-	-	-	+	+
AP1TI2	Solid ground	Winter	+	Diplobacilli	-	-	-	-	-	+	-	+	-
AP1TI3	Solid ground	Winter	+	Bacilli	-	+	-	-	-	+	-	+	+
AP1TI4	Solid ground	Winter	+	Diplobacilli	-	-	-	-	-	-	-	+	+
AP2TI1	Solid ground	Winter	+	Diplobacilli	-	-	-	-	-	-	-	+	+
AP2TI2	Solid ground	Winter	+	Bacilli	+	-	-	-	-	-	-	+	+
AP2TI3	Solid ground	Winter	+	Diplobacilli	-	-	-	-	-	-	-	+	-
AP2TI4	Solid ground	Winter	+	Diplobacilli	-	-	-	-	-	+	-	+	+
AP2TI5	Solid ground	Winter	-	Coccobacillus	-	+	+	+	-	+	+	-	-
AP3TI1	Solid ground	Winter	+	Diplobacilli	-	-	-	+	-	-	-	+	+
AP3TI2	Solid ground	Winter	+	Bacilli	+	-	-	-	-	-	-	+	-
AP3TI3	Solid ground	Winter	+	Bacilli	+	-	-	-	-	-	-	+	+

AP3TI4	Solid ground	Winter	+	Coccobacillus	-	-	-	-	-	-	-	-	-
AP3TI5	Solid ground	Winter	+	Coccobacillus	-	-	-	-	-	-	-	+	-
AP3TI6	Solid ground	Winter	+	Bacilli	+	-	-	-	-	-	-	+	-
AP4TI1	Solid ground	Winter	+	Diplobacilli	-	-	-	-	-	+	-	+	-
AP4TI2	Solid ground	Winter	+	Bacilli	-	-	-	-	-	+	-	+	+
AP4TI3	Solid ground	Winter	+	Diplobacilli	-	-	-	-	-	-	-	+	-
AP4TI4	Solid ground	Winter	-	Coccos	+	-	-	-	-	-	-	-	-
AP1VI1	Floodplain	Winter	+	Bacilli	-	-	-	-	-	+	-	+	-
AP1VI2	Floodplain	Winter	+	Diplobacilli	-	-	-	-	-	-	-	+	-
AP1VI3	Floodplain	Winter	-	Coccobacillus	+	+	+	+	-	-	+	+	-
AP1VI4	Floodplain	Winter	+	Diplobacilli	-	-	-	-	-	-	-	+	-
AP1VI5	Floodplain	Winter	+	Bacilli	+	-	-	-	-	-	-	+	+
AP2VI1	Floodplain	Winter	+	Diplobacilli	-	-	-	-	-	-	-	+	-
AP2VI2	Floodplain	Winter	+	Streptobacilli	-	-	-	-	-	-	-	+	-
AP2VI3	Floodplain	Winter	+	Bacilli	-	-	-	-	-	-	-	+	-
AP2VI4	Floodplain	Winter	+	Streptobacilli	-	-	-	-	-	-	-	+	+
AP2VI5	Floodplain	Winter	+	Diplobacilli	-	-	-	-	-	-	-	+	-
AP2VI6	Floodplain	Winter	+	Streptobacilli	-	-	-	-	-	-	-	+	-
AP2VI7	Floodplain	Winter	-	Coccobacillus	-	+	+	+	-	+	+	+	-
AP2VI8	Floodplain	Winter	+	Diplobacilli	+	-	-	-	-	-	-	+	+
AP3VI1	Floodplain	Winter	+	Streptobacilli	-	-	-	-	-	+	-	+	-
AP3VI2	Floodplain	Winter	+	Diplobacilli	-	-	-	-	-	+	-	+	-
AP3VI3	Floodplain	Winter	+	Diplobacilli	-	-	-	-	-	-	-	+	-
AP3VI4	Floodplain	Winter	+	Diplobacilli	+	-	-	-	-	-	-	+	-
AP4VI1	Floodplain	Winter	+	Diplobacilli	-	-	-	-	-	-	-	+	-
AP4VI2	Floodplain	Winter	+	Bacilli	-	-	-	-	-	-	-	+	-

AP4VI3	Floodplain	Winter	+	Bacilli	+	-	-	-	-	+	-	+	-
AP4VI4	Floodplain	Winter	+	Diplobacilli	-	-	-	-	-	-	-	+	-
AP4VI5	Floodplain	Winter	+	Diplobacilli	-	-	-	-	-	-	-	+	+
AP4VI6	Floodplain	Winter	-	Spirochete	-	-	-	-	-	-	-	+	-
AP4VI7	Floodplain	Winter	+	Bacilli	-	-	+	-	-	+	-	+	-
AP4VI8	Floodplain	Winter	+	Streptobacilli	-	-	-	-	-	-	-	+	-
AP4VI9	Floodplain	Winter	+	Diplobacilli	-	-	-	-	-	-	-	+	-
AP4VI10	Floodplain	Winter	+	Diplobacilli	-	-	-	-	-	-	-	+	-
AP1VI^{A}1	Floodplain	Winter	+	Bacilli	-	-	-	-	-	-	-	+	+
AP1VI^{A}2	Floodplain	Winter	+	Streptobacilli	-	-	-	-	-	-	-	+	+
AP1VI^{A}3	Floodplain	Winter	+	Streptobacilli	-	-	-	-	-	-	-	+	+
AP1VI^{A}4	Floodplain	Winter	+	Bacilli	-	-	-	-	-	-	-	+	+
AP1VI^{A}5	Floodplain	Winter	+	Bacilli	-	-	-	+	-	-	-	+	+
AP1VI^{A}6	Floodplain	Winter	+	Streptobacilli	-	-	-	-	-	-	-	+	+
AP1VI^{A}7	Floodplain	Winter	+	Bacilli	-	-	-	-	-	-	-	+	-
AP1VI^{A}8	Floodplain	Winter	-	Coccobacillus	-	-	-	+	-	-	-	+	+
AP1VI^{A}9	Floodplain	Winter	+	Bacilli	-	-	-	-	-	-	-	+	+
AP1VI^{A}10	Floodplain	Winter	+	Bacilli	-	-	-	-	-	-	-	+	+
AP1VI^{A}11	Floodplain	Winter	+	Streptobacilli	-	-	-	-	-	-	-	+	+
AP1VI^{A}12	Floodplain	Winter	+	Bacilli	-	-	-	-	+	+	-	+	+
AP1VI^{A}13	Floodplain	Winter	+	Bacilli	-	-	-	-	-	-	-	+	+
AP2VI^{A}1	Floodplain	Winter	+	Diplobacilli	-	-	-	-	-	-	-	+	+
AP2VI^{A}2	Floodplain	Winter	+	Streptobacilli	-	-	-	-	-	-	-	+	+
AP2VI^{A}3	Floodplain	Winter	+	Diplobacilli	-	-	-	-	-	-	-	+	+
AP2VI^{A}4	Floodplain	Winter	+	Bacilli	-	-	-	-	-	-	-	+	+
AP2VI^{A}5	Floodplain	Winter	+	Bacilli	-	-	-	-	-	-	-	+	+

AP2VI^{A}6	Floodplain	Winter	+	Bacilli	-	-	-	+	-	-	-	+	+
AP2VI^{A}7	Floodplain	Winter	+	Bacilli	-	-	-	-	-	-	-	+	-
AP2VI^{A}8	Floodplain	Winter	+	Streptobacilli	-	-	-	-	+	-	-	+	+
AP3VI^{A}1	Floodplain	Winter	+	Diplobacilli	-	-	-	-	-	-	-	-	+
AP3VI^{A}2	Floodplain	Winter	+	Bacilli	-	-	-	-	-	-	-	+	+
AP3VI^{A}3	Floodplain	Winter	+	Diplobacilli	-	-	-	-	-	-	-	+	-
AP3VI^{A}4	Floodplain	Winter	+	Bacilli	-	-	-	-	-	-	-	+	-
AP3VI^{A}5	Floodplain	Winter	+	Streptobacilli	-	-	-	-	-	-	-	+	+
AP3VI^{A}6	Floodplain	Winter	+	Diplobacilli	-	-	-	-	-	-	-	+	+
AP3VI^{A}7	Floodplain	Winter	+	Diplobacilli	-	-	-	-	-	-	-	+	+
AP3VI^{A}8	Floodplain	Winter	+	Diplobacilli	+	-	-	-	-	-	-	+	+
AP3VI^{A}9	Floodplain	Winter	+	Bacilli	-	-	-	-	-	-	-	-	-
AP3VI^{A}10	Floodplain	Winter	+	Diplobacilli	-	-	-	-	-	-	-	+	+
AP3VI^{A}11	Floodplain	Winter	+	Bacilli	-	-	-	-	-	-	-	+	+
AP3VI^{A}12	Floodplain	Winter	+	Diplobacilli	+	-	-	-	-	-	-	+	+
AP4VI^{A}1	Floodplain	Winter	+	Bacilli	+	-	-	-	-	-	-	+	+
AP4VI^{A}2	Floodplain	Winter	+	Bacilli	-	-	-	-	-	-	-	+	+
AP4VI^{A}3	Floodplain	Winter	+	Diplobacilli	-	-	-	-	-	-	-	+	-
AP4VI^{A}4	Floodplain	Winter	+	Diplobacilli	-	-	-	-	-	-	-	+	-
AP4VI^{A}5	Floodplain	Winter	+	Coccobacillus	-	-	-	-	-	-	-	+	+

Legend: (+) positive result; (-) negative result; (AMS) Antimicrobial substances; (ACC) 1-aminocyclopropane-1-carboxylate; (IAA) indole-3-acetic acid.
