# Supplementary figures and images for: Rhizobacteria Isolated from Amazonian Soils Reduce the Effects of Water Stress on the Growth of Açaí (Euterpe oleracea Mart.) Palm Seedlings
Source: Biology (Basel). 2024 Sep 24;13(10):757. doi: 10.3390/biology13100757 (PMC11504209; doi:10.3390/biology13100757)

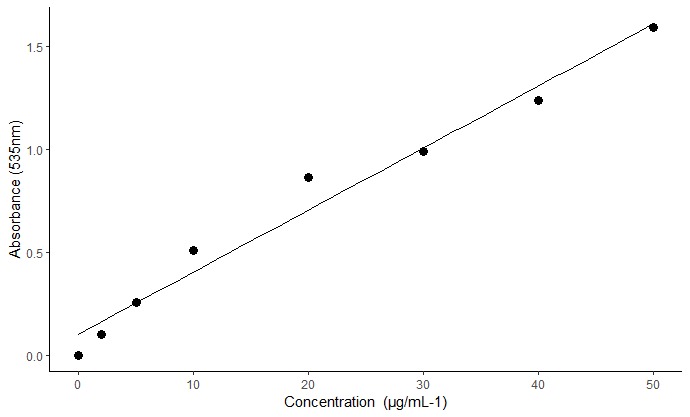

Supplement: Supplementary file 1 [file biology-13-00757-s001.zip › Figure S1.jpeg]
